# STOX1 deficiency is associated with renin-mediated gestational hypertension and placental defects

**DOI:** 10.1172/jci.insight.141588

**Published:** 2021-01-25

**Authors:** Jacqueline G. Parchem, Keizo Kanasaki, Soo Bong Lee, Megumi Kanasaki, Joyce L. Yang, Yong Xu, Kadeshia M. Earl, Rachel A. Keuls, Vincent H. Gattone, Raghu Kalluri

**Affiliations:** 1 Department of Cancer Biology, Metastasis Research Center, University of Texas MD Anderson Cancer Center, Houston, Texas, USA.; 2 Department of Obstetrics, Gynecology and Reproductive Sciences, McGovern Medical School, The University of Texas Health Science Center at Houston, Texas, USA.; 3 Division of Matrix Biology, Department of Medicine, Beth Israel Deaconess Medical Center and Harvard Medical School, Boston, Massachusetts, USA.; 4 Development, Disease Models & Therapeutics Graduate Program, Center for Cell and Gene Therapy, and Stem Cells and Regenerative Medicine Center, Department of Molecular and Cellular Biology, Baylor College of Medicine, Houston, Texas, USA.; 5 Department of Anatomy and Cell Biology, Indiana University School of Medicine, Indianapolis, Indiana, USA.

**Keywords:** Cardiology, Reproductive Biology, Hypertension, Mouse models, Obstetrics/gynecology

## Abstract

The pathogenesis of preeclampsia and other hypertensive disorders of pregnancy remains poorly defined despite the substantial burden of maternal and neonatal morbidity associated with these conditions. In particular, the role of genetic variants as determinants of disease susceptibility is understudied. Storkhead-box protein 1 (*STOX1*) was first identified as a preeclampsia risk gene through family-based genetic linkage studies in which loss-of-function variants were proposed to underlie increased preeclampsia susceptibility. We generated a genetic *Stox1* loss-of-function mouse model (Stox1 KO) to evaluate whether STOX1 regulates blood pressure in pregnancy. Pregnant Stox1-KO mice developed gestational hypertension evidenced by a significant increase in blood pressure compared with WT by E17.5. While severe renal, placental, or fetal growth abnormalities were not observed, the Stox1-KO phenotype was associated with placental vascular and extracellular matrix abnormalities. Mechanistically, we found that gestational hypertension in Stox1-KO mice resulted from activation of the uteroplacental renin-angiotensin system. This mechanism was supported by showing that treatment of pregnant Stox1-KO mice with an angiotensin II receptor blocker rescued the phenotype. Our study demonstrates the utility of genetic mouse models for uncovering links between genetic variants and effector pathways implicated in the pathogenesis of hypertensive disorders of pregnancy.

## Introduction

Hypertensive disorders of pregnancy represent a spectrum of diagnoses thought to originate from abnormal placentation, which causes vascular dysfunction and a maternal inflammatory response (1–3). The maternal and neonatal morbidity and health care costs associated with these conditions are substantial (4, 5). Nevertheless, meaningful advances in the management of preeclampsia and other hypertensive disorders of pregnancy have been hampered by an incomplete understanding of disease pathophysiology.

Current evidence indicates that the preeclampsia syndrome of hypertension and end organ injury is the manifestation of a complex disorder resulting from multiple different causes (2, 3). Mouse models have been critical for uncovering the roles of specific genetic factors in pregnancy-related hypertension (6–10). The majority of these models, however, result in severe phenotypes, which do not recapitulate the variation in clinical presentations of pregnancy-related hypertensive disorders. Thus, there is a gap in our knowledge of the molecular underpinnings of more common, and potentially insidious, clinical entities on the “milder” end of the disease spectrum, such as gestational hypertension.

One area of uncertainty is the role of specific genetic variants as determinants of disease susceptibility, although genome-wide association studies and efforts to screen functionally relevant biomarkers in human pregnancy are ongoing (11–17). In 2005, van Dijk and colleagues linked the gene encoding the Storkhead-box protein 1 transcription factor (*STOX1*) to preeclampsia and gestational hypertension in Dutch families (18). The authors reported a common, maternally inherited single nucleotide variant, Y153H, predicted to alter the function of the DNA-binding domain, and proposed that loss of STOX1 function in the placenta was the underlying cause of preeclampsia in their cohort. Subsequent studies challenged the conclusions of the study (19, 20), including the robustness of the association between Y153H and preeclampsia given the prevalence of this variant among unaffected pregnancies (20–23). Although additional studies have examined the function of STOX1 (24–27), the original hypothesis that STOX1 loss of function increases the risk of hypertension in pregnancy has not been tested to our knowledge.

To ascertain the role of STOX1 in regulating gestational blood pressure, we generated and characterized a genetic loss-of-function mouse model (Stox1 KO). We show that pregnancies resulting from breeding of homozygous Stox1-KO mice develop gestational hypertension, without overt signs of preeclampsia. Mechanistically, our data show that the hypertensive phenotype in Stox1-KO mice resulted from upregulation of the uteroplacental renin-angiotensin system (RAS), which was alleviated by antagonizing the effects of renin with angiotensin II receptor blocker therapy. Stox1-KO placentas were associated with increased decidual vascularization and extracellular matrix deposition, in response to tissue hypoxia. Analysis of published single-cell RNA sequencing (scRNA-Seq) data from E9.5 mouse placenta showed that *Stox1*^+^ cells express high levels of endothelial cell markers, suggesting a role for STOX1 in regulating placental vascular development. An investigation of the human population frequency *STOX1* variants revealed that Y153H is the most frequently detected variant, suggesting that it represents a smaller effect size, common variant, rather than a deleterious, loss-of-function variant. Collectively, our results show that STOX1 negatively regulates renin expression in the placenta and are consistent with the well-supported premise that common conditions are influenced by variants in many genetic loci.

## Results

### Generation of Stox1-KO mice and normal Stox1 expression.

**Figure 1:**
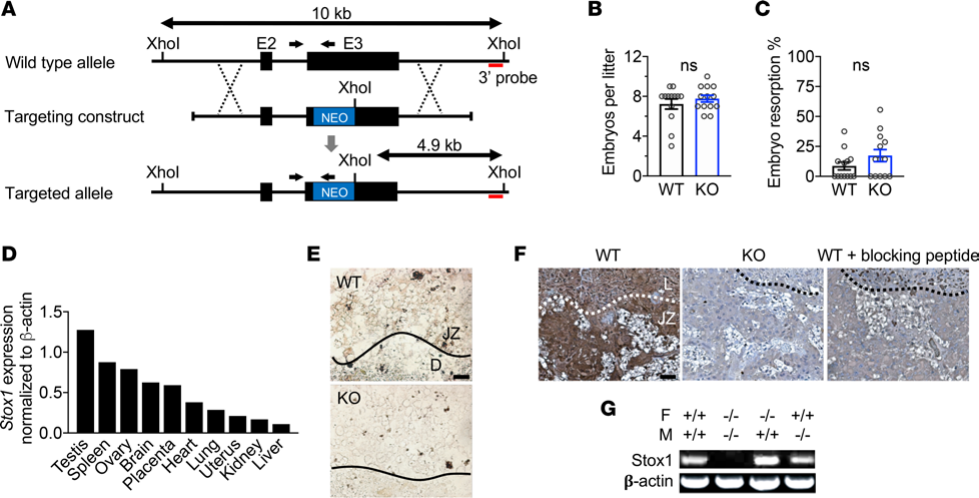
Generation of Stox1-KO mouse and placental expression of Stox1. (**A**) Targeting strategy used to generate Stox1-KO mice containing an approximately 1 kb deletion of exon 3. (**B** and **C**) Total number of embryos (**B**) and (**C**) percentage of resorbed embryos per litter (*n* = 13 litters). (**D**) *Stox1* mRNA expression in various tissues by reverse transcription PCR (RT-PCR), normalized to β-actin. (**E** and **F**) In situ hybridization (**E**) and immunohistochemistry (**F**) showing normal Stox1 expression in WT placenta and lack of expression in KO. Scale bars: 50 μm. D, decidua; JZ, junctional zone; L, labyrinth. (**G**) Biallelic expression of *Stox1* mRNA in the placenta, evidenced by expression from either maternal or paternal allele in heterozygous placentas (RT-PCR). *Stox1* genotypes of female and male mice shown. F, female; M, male. Results are shown as mean ± SEM. Two-tailed, unpaired *t* test.

The *Stox1* gene was inactivated by targeted disruption of exon 3 (Figure 1A) and confirmed by Southern blot analysis and PCR (Supplemental Figure 1, A and B; supplemental material available online with this article; https://doi.org/10.1172/jci.insight.141588DS1). *Stox1* heterozygous crosses produced Stox1-KO offspring at the expected Mendelian ratio. Stox1-KO and WT lines were maintained by breeding homozygous KO and WT mice, respectively. Differences in litter size and embryo resorption were not observed in Stox1-KO compared with WT pregnancies (Figure 1, B and C). In WT mice, *Stox1* mRNA was detected in several tissues, including the placenta (Figure 1D and Supplemental Figure 1C). Placental expression was confirmed by in situ hybridization (Figure 1E) and antibody staining (Figure 1F). Biallelic expression of *Stox1* was also observed (Figure 1G), consistent with data suggesting that human *STOX1* is not imprinted (20).

### Stox1-KO mice develop gestational hypertension.

**Figure 2:**
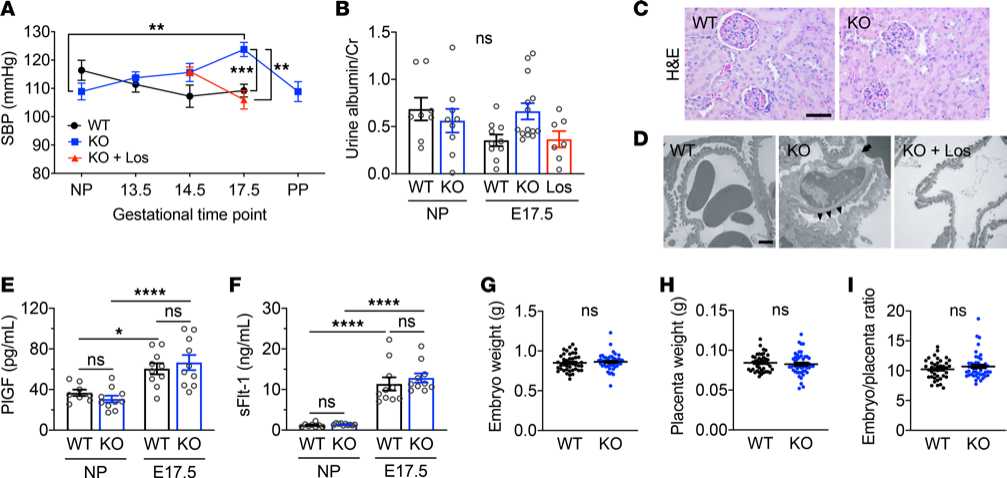
Loss of STOX1 results in gestational hypertension. (**A**) Systolic blood pressure at different time points for WT and Stox1-KO cohorts. A subset of Stox1-KO, pregnant mice were treated daily with losartan (KO + Los) starting at E14.5 until sacrifice. NP, nonpregnant; SBP, systolic blood pressure; PP, postpartum (day 10). Details including mouse numbers and all comparisons are in Supplemental Table 1. (**B**) Urine albumin/creatinine ratio for NP (WT, *n* = 8; KO, *n* = 9) and pregnant mice at E17.5 (WT, *n* = 10; KO, *n* = 14; KO + Los, *n* = 7). (**C**) Representative images of H&E-stained maternal kidney sections from the indicated groups showing normal tubules and glomeruli. Scale bar: 50 μm. (**D**) Transmission electron micrographs of glomeruli. Arrowheads point to swollen podocyte foot processes; arrow points to exuberant endothelial cells with processes extending into the lumen in Stox1 KO. Scale bar: 100 nm. (**E** and **F**) Serum PlGF (**E**) and sFlt-1 (**F**) levels measured by ELISA in nonpregnant WT (*n* = 8) and Stox1 KO (*n* = 10) and pregnant WT and KO at E17.5 (*n* = 10). (**G** and **H**) Similar weights of embryos (**G**) and placentas (**H**) from WT (*n* = 41 embryos/placentas from 7 litters) and KO (*n* = 47 embryos/placentas from 8 litters) collected at E17.5. (**I**) Similar embryo/placenta ratio in WT and KO. Data are mean ± SEM. One-way ANOVA with Bonferroni’s correction (**A**, **B**, **E**, and **F**); 2-tailed, unpaired *t* test (**G**–**I**). Adjusted *P ≤ 0.05, **P ≤ 0.01, ***P ≤ 0.001, ****P ≤ 0.0001.

To determine the role of STOX1 in regulating gestational blood pressure, we obtained serial blood pressure measurements across gestation (Figure 2A, Supplemental Table 1). For WT mice, systolic blood pressures during pregnancy were lower than nonpregnant baseline. In contrast, blood pressures increased with gestational age among pregnant Stox1-KO mice and were significantly elevated in Stox1-KO mice at E17.5 compared with WT pregnant mice at E17.5 (124 ± 10 vs. 109 ± 9, adjusted *P* ≤ 0.01) and nonpregnant Stox1 KO. Postpartum, blood pressure normalization was observed in Stox1-KO mice.

We further assessed pregnant mice for renal signs of preeclampsia: proteinuria and glomerular endotheliosis (28). Significant differences in proteinuria were not observed between the nonpregnant and pregnant (E17.5) states or between WT and KO mice (Figure 2B). Histologic analysis of the maternal kidney did not reveal apparent differences in KO versus WT by H&E (Figure 2C). Electron micrographs of the glomerular filtration barrier of KO kidneys showed rare areas containing edematous podocyte foot processes and prominent endothelial cells but were without evidence of substantial podocyte foot process effacement or glomerular endotheliosis (Figure 2D).

We next analyzed serum levels of placental growth factor (PlGF) and soluble fms-like tyrosine kinase-1 (sFlt-1), which are consistently altered in preeclampsia (reduced PlGF, elevated sFlt-1) and reflective of underlying placental dysfunction (29). Both biomarkers increased during pregnancy as expected in WT and-KO mice (Figure 2, E and F). However, differences in PlGF and sFlt-1 in pregnancy were not observed between WT and KO. To further examine placental function, weights of embryos and placentas at E17.5 were compared and found to be similar for KO compared with WT litters (Figure 2, G and H). The mean embryo/placenta ratio, a proxy for placental function (30), was also similar between groups (Figure 2I).

### Activation of the uteroplacental RAS in Stox1-KO mice.

**Figure 3:**
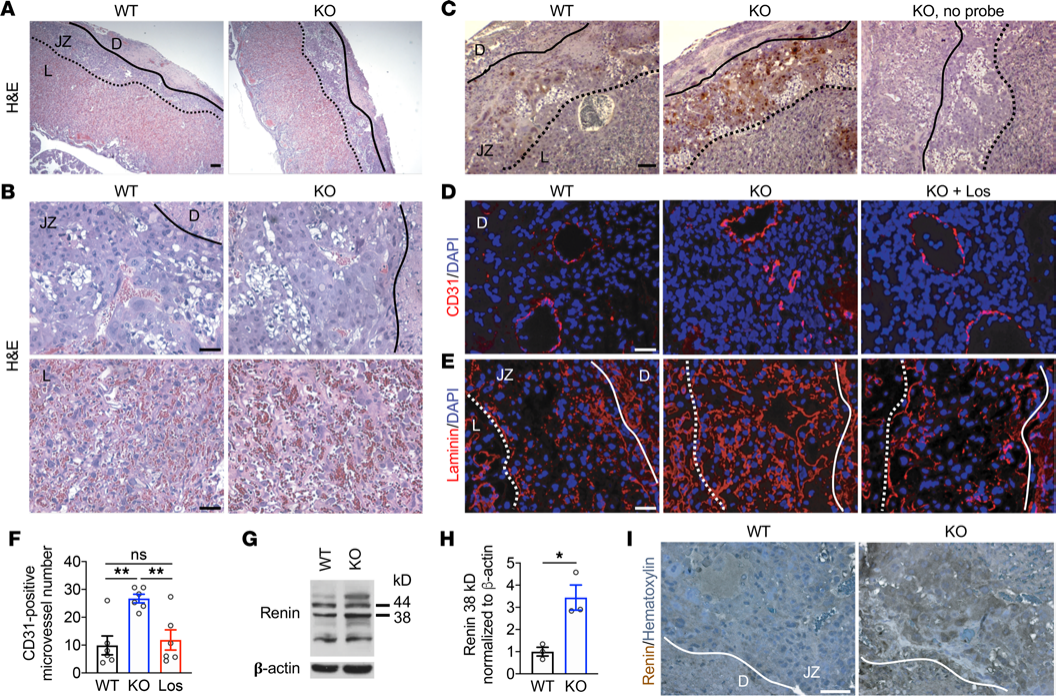
Hypoxia and renin upregulation in Stox1-KO placentas. (**A**) Representative low-magnification images of H&E-stained WT and KO placenta at E17.5. Scale bar: 200 μm. (**B**) High-magnification images of the junctional zone and labyrinth. Scale bar: 50 μm. (**C**) Hypoxyprobe immunohistochemistry of the placenta from WT, KO, and no probe control shows increased hypoxia in the junctional zone of the KO. Scale bar: 100 μm. (**D**) CD31 immunofluorescence identifies blood vessels in the decidua in WT, KO, and KO treated with losartan (KO + Los) groups at E17.5. Scale bar: 50 μm. (**E**) Increased junctional zone laminin deposition in KO compared with WT and KO + Los. Scale bar: 50 μm. (**F**) Quantification of CD31-positive microvessels in **D** in WT, KO, and KO + losartan placentas (*n* = 6 placentas from 3–4 litters, 3 high-power fields per placenta). (**G**) Renin expression in the placenta by Western blot. (**H**) Quantification of active renin 38 kDa peptide in replicate blots (*n* = 3). (**I**) Immunohistochemistry for placental renin. Scale bar: 50 μm. D, decidua; JZ, junctional zone; L, labyrinth. Solid line marks border between the decidua and junctional zone; dotted line marks border between junctional zone and labyrinth. Data are shown as mean ± SEM. One-way ANOVA with Bonferroni’s correction (**F**); 2-tailed, unpaired *t* test (**H**). *P ≤ 0.05, **adjusted *P* ≤ 0.01.

Given the central role of the placenta in hypertensive disorders of pregnancy, we performed histologic analyses of the placenta. No apparent differences in placental architecture (Figure 3A) or trophoblast morphology in the junctional zone or labyrinth layers (Figure 3B) were observed in Stox1-KO compared with WT placentas (E17.5) stained with H&E. To examine the possibility of impaired placental perfusion, which is characteristic of gestational hypertension, we injected hypoxyprobe into pregnant mice at E17.5 just before sacrifice to visualize regions of tissue hypoxia. KO placentas showed increased staining for hypoxyprobe in the junctional zone compared with WT (Figure 3C), indicating some degree of placental hypoxia in Stox1-KO mice by late gestation.

We further analyzed the decidual microvasculature and extracellular matrix deposition, as changes in both have been associated with placental hypoxia (31–33). A higher density of CD31-positive vessels was observed in the decidua of KO placentas compared with WT (Figure 3, D and F). Staining for extracellular matrix proteins laminin, nidogen-1, and type IV collagen α1 was also increased in the junctional zone and decidua of Stox1-KO placentas (Figure 3E and Supplemental Figure 2A).

To investigate the molecular mechanisms underlying the Stox1-KO phenotype, we examined the placental expression of genes previously implicated in preeclampsia pathogenesis (Supplemental Figure 2B). Several genes, including *Edn1* (Endothelin), *Tnf* (TNF-α), *Nos2* (iNOS), and *Eng* (Endoglin), were expressed at similar levels in Stox1-KO and WT placentas. KO placentas did, however, show a marked increase in *Ren1* (renin) expression (Supplemental Figure 2, B and C), a core enzyme of the RAS. Renin indirectly increases levels of angiotensin II (Ang II), which in turn increases blood pressure by promoting vasoconstriction and stimulating aldosterone secretion. Although the kidney is the major producer of circulating RAS factors, in pregnancy, an independent, local uteroplacental RAS exists (34–36).

Increased placental renin mRNA expression was observed only in Stox1-KO placentas (Supplemental Figure 2D). Higher levels of placental renin were confirmed by detection of cleaved, active renin (38 kDa) (37) on Western blot (Figure 3, G and H) and by renin immunohistochemistry (Figure 3I). Assessment of renin expression in the kidney did not reveal an increase in active renin in KO kidneys by RT-PCR or Western blot (Supplemental Figure 2, E and F), suggesting that STOX1 specifically regulates placental renin.

To validate RAS activation as the mechanism underlying gestational hypertension in Stox1-KO mice, we treated pregnant mice with the Ang II receptor blocker (ARB) losartan. Losartan therapy starting at E14.5 rescued the Stox1-KO phenotype, normalizing blood pressure to values comparable to WT (Figure 2A). Treatment of pregnant WT mice with losartan did not further lower blood pressure below untreated WT (data not shown). Although losartan treatment was not associated with a significant reduction in proteinuria (Figure 2B), treated Stox1-KO mice had normal-appearing glomeruli (Figure 2D) and amelioration of microscopic vascular and basement membrane abnormalities observed in untreated KO placentas (Figure 3, D–F, and Supplemental Figure 2A).

### Stox1^+^ cells in the early placenta express endothelial cell markers.

**Figure 4:**
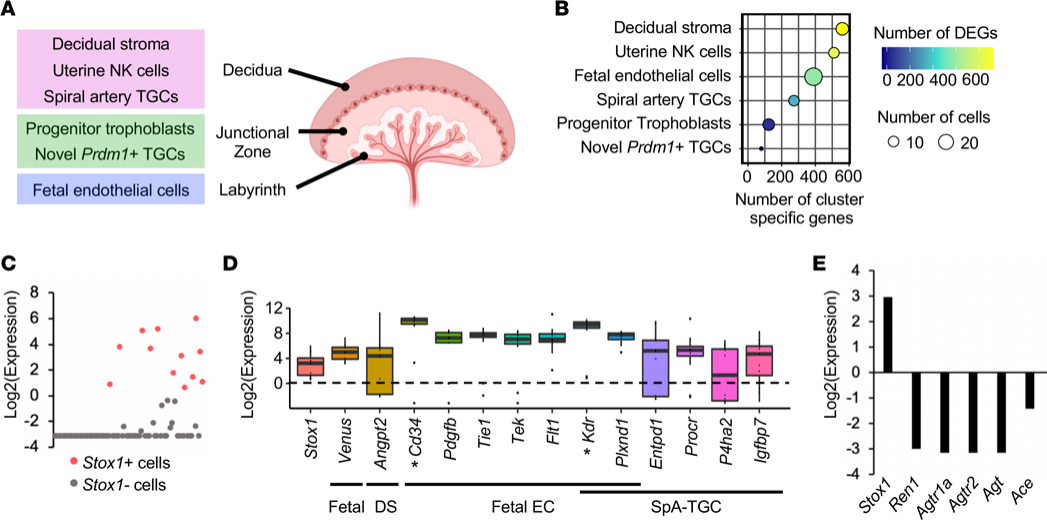
Analysis of scRNA-Seq data from E9.5 mouse placenta shows coexpression of *Stox1* with endothelial cell and SpA-TGC markers. (**A**) Diagram showing 6 cell clusters defined in data set from Nelson et al. (38). (**B**) Overview of the number of variable features producing 6 cell populations obtained from the analyzed samples. (**C**) Scatter plot showing *Stox1* expression in profiled single cells. Population of *Stox1*^+^ cells analyzed in **D** and **E** shown in red. (**D**) Selected cell population markers expressed in *Stox1*^+^ cells. Asterisks mark *Kdr* and *Cd34*, which were also markers of the novel *Prdm1*^+^ TGC population. (**E**) Expression of renin-angiotensin pathway members in *Stox1*^+^ cells. DEGs, differentially expressed genes; DS, decidual stroma; EC, endothelial cell; NK, natural killer; SpA-TGCs, spiral artery trophoblast giant cell; TGC, trophoblast giant cell. Data are normalized expression values: per cell (**C**), median ± IQR (**D**), or mean (**E**).

The upward trend in maternal blood pressure observed by approximately E14.5 in the Stox1 KO (Figure 2A) led us to hypothesize that STOX1 plays a key role in the placenta earlier in gestation. Thus, we analyzed published scRNA-Seq data to glean insight into the identity of *Stox1*^+^ cells (38). In the original study, Nelson and colleagues profiled *Prdm1*/Blimp1^+^ single cells from E9.5 mouse placenta. *Prdm1* encodes a transcription factor that defines a broad range of cells in the developing placenta: spiral artery trophoblast giant cells (SpA-TGCs) and other maternal cells within the decidua, glycogen trophoblasts and proliferative diploid trophoblasts in the junctional zone, and fetal endothelial cells in the labyrinth (38, 39). The original study identified 6 unique cell clusters: 1) decidual stroma, 2) uterine natural killer cells, 3) SpA-TGCs, 4) novel *Prdm1*^+^ TGCs (putative parietal TGC precursor), 5) progenitor trophoblasts, and 6) fetal endothelial cells (Figure 4, A and B).

We analyzed the subset of *Stox1*^+^ cells (15% of profiled cells; Figure 4C) for the expression of the genes used to define the 6 clusters in Nelson et al. All *Stox1*^+^ cells expressed the paternally derived *Venus* transgene used to identify fetal cells (Figure 4D). Notably, *Stox1*^+^ cells expressed a number of vascular/endothelial cell surface markers (40), including *Cd34*, *Plxnd1*, angiopoietin receptors *Tie1* and *Tek/Tie2*, and VEGF receptors *Flt1* and *Kdr*/*Flk1* (Figure 4D). These markers were used to define the fetal endothelial cell cluster; however, some markers were also expressed in other clusters: SpA-TGCs (*Flt1*, *Kdr/Flk1*, *Plxnd1*), which remodel maternal spiral arteries and engage in vascular mimicry (41); and the novel *Prdm1*^+^ TGC/precursor parietal TGC cluster (*Kdr/Flk1*, *Cd34*) (38). Other vascular genes marking SpA-TGCs were also expressed in *Stox1*^+^ cells, such as *Entpd1* and *Procr*. Some *Stox1*^+^ cells (albeit a very small number) expressed high levels of progenitor trophoblast markers (Supplemental Figure 3A). Together, these data suggest that *Stox1* loss in the early placenta primarily affects vascular cells with fetal origin, namely fetal endothelial cells of the labyrinth, which form from the allantois (42), and endovascular SpA-TGCs in the decidua. Consistent with our in vivo data showing STOX1 suppression of the RAS, downregulation of renin-angiotensin pathway genes (*Ren1*, *Agtr1a*, *Agtr2*, *Agt*, and *Ace*), was noted in *Stox1*^+^ cells (Figure 4E), lending further evidence to support the proposed mechanism for the KO phenotype.

**Figure 5:**
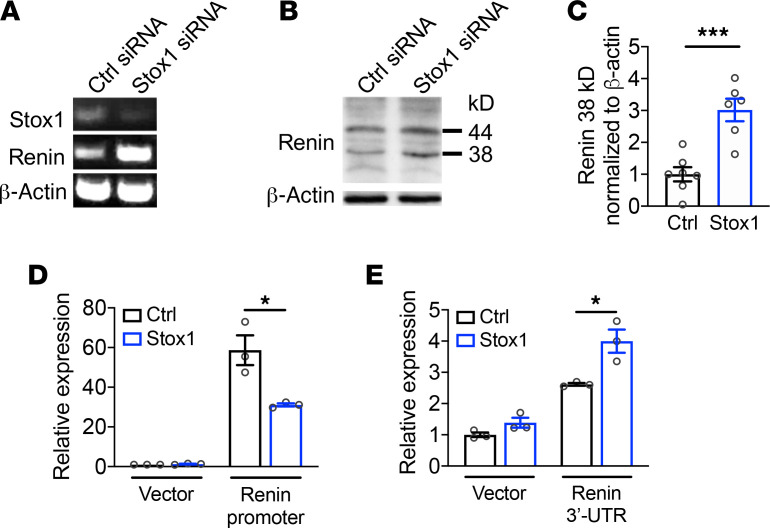
STOX1 regulates renin expression in human cytotrophoblasts. (**A**) siRNA knockdown of *STOX1* in HTR-8/SVneo cells results in increased renin transcription (RT-PCR). (**B**) Increased active renin peptide (38 kDa) with *STOX1* knockdown by Western blot. (**C**) Quantification of replicate Western blots, normalized to β-actin (*n* = 7; 1 outlier identified by ROUT method excluded from Stox1 siRNA group). (**D**) Dual-luciferase assay of HTR-8/SVneo cells transfected with empty luciferase vector control or human *REN* promoter-luciferase construct. Baseline renin promoter-luciferase activity detected in control siRNA condition. *STOX1* siRNA knockdown resulted in decreased renin promoter activity, suggesting negative feedback regulation by renin. Relative luciferase expression normalized to vector control with Ctrl siRNA (*n* = 3). (**E**) Human renin 3′-UTR-luciferase reporter showing increased reporter activity with *Stox1* knockdown suggesting that STOX1 repression of renin involves the 3′-UTR (*n* = 3). Ctrl, control. Data are shown as mean ± SEM. Two-tailed, unpaired *t* test. *P ≤ 0.05, ***P ≤ 0.001.

*STOX1 suppresses renin via the 3′-UTR*. Our mouse data suggested that STOX1 normally suppresses renin in the placenta. To investigate this interaction, we performed siRNA knockdown of *STOX1* in the human cytotrophoblast cell line HTR-8/SVneo (43), which contains the *STOX1* Y153H variant allele (Supplemental Figure 4). *STOX1* knockdown resulted in increased *REN* (renin) mRNA (Figure 5A) and active renin peptide (38 kDa; Figure 5, B and C), consistent with the in vivo data.

To determine if STOX1 negatively regulates the renin promoter, we generated a luciferase reporter construct containing the human renin promoter. HTR-8 cells transfected with vector control showed negligible baseline luciferase activity (Figure 5D). Transfection of the renin promoter reporter construct with control siRNA showed robust expression of luciferase, indicating baseline activation of the renin promoter in HTR-8 cells. In contrast, transfection of the reporter with Stox1 siRNA resulted in suppression of renin promoter activity, when renin levels are elevated. Thus STOX1 does not repress the renin promoter; rather, renin promoter activity appeared to be responsive to local renin levels, consistent with negative feedback regulation.

Next, we tested an alternative mechanism of posttranscriptional regulation of the renin 3′-UTR. We generated luciferase constructs containing the 3′-UTR of human renin, with and without a constitutive CMV promoter. Transfection of the control construct without the CMV promoter showed minimal basal luciferase activity (Figure 5E). Transfection of the CMV-luciferase-renin-3′-UTR construct resulted in robust luciferase activity in the control siRNA condition. Luciferase activity was further increased with knockdown of *STOX1*. Thus, inhibition of *STOX1* relieved repression of the renin 3′-UTR. Our results suggest that STOX1 regulates placental renin expression via a posttranscriptional mechanism involving the renin 3′-UTR.

### STOX1 is a conserved transcription factor with common single nucleotide variants.

**Figure 6:**
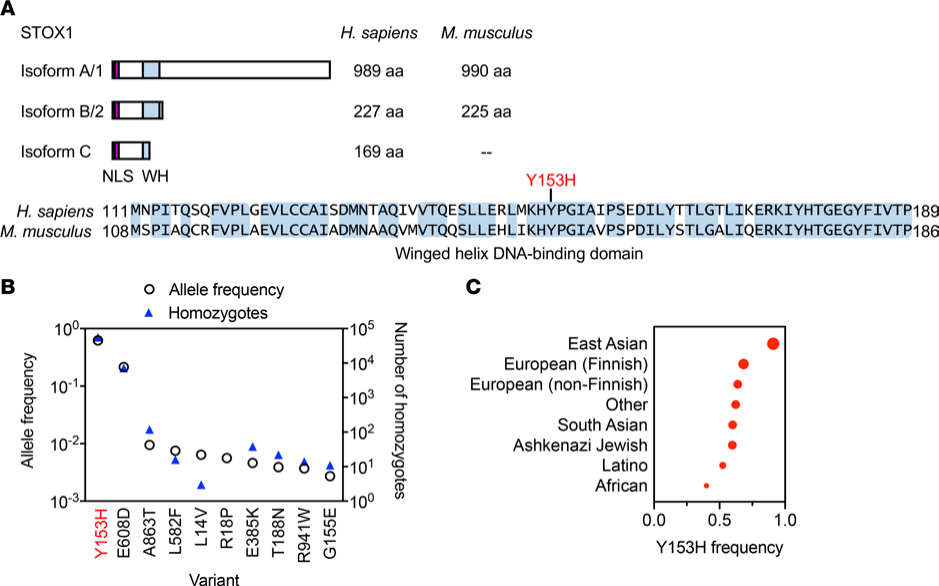
STOX1 is conserved and Y153H is the most common variant in population data. (**A**) Comparison of STOX1 isoforms in human and mouse with sequence alignment of WH DNA-binding domain show high sequence identity (shaded blue). Location of Y153H variant marked. NLS, nuclear localization signal; WH, winged helix. (**B**) Frequency and number of homozygotes for the 10 most common missense variants in *STOX1* and (**C**) population frequencies of the Y153H variant from gnomAD.

To examine the potential relevance of our results to humans, we aligned the amino acid sequences of the dominant isoforms of mouse (isoform 1) and human (isoform A) STOX1 (Figure 6A). The alignment revealed high sequence similarity for the full-length protein (59.7% identity, 80.0% similarity) and showed that the DNA-binding winged helix (WH) domain was highly conserved (78.5% identity, 98.7% similarity). Data are emerging on the functional relevance of other truncated isoforms (44, 45).

We also investigated the population frequency of the Y153H variant, which resides in the WH domain (Figure 6A) and was originally implicated in preeclampsia susceptibility (18). We searched the Genome Aggregation Database (gnomAD), a large population reference containing data aggregated from 125,748 exomes and 15,708 genomes (46). Of 438 (51%) missense single nucleotide variants in *STOX1* (Table 1), Y153H was most frequently detected (allele frequency 0.62; Figure 6B). In addition, over 55,000 individuals homozygous for Y153H were identified in the data set. Although previous studies focused on European cohorts, Y153H was detected in ethnically diverse population subgroups (Figure 6C). There were no deviations in the observed-to-expected ratios for missense and predicted loss-of-function variants, suggesting that STOX1 loss of function is not deleterious in humans (Table 1).

## Discussion

Our data show that STOX1 loss of function in mice causes gestational hypertension resulting from uteroplacental RAS activation, which is reversed with ARB therapy. Stox1-KO mice lacked overt signs of preeclampsia, despite evidence of placental hypoxia, and developed hypertension late in gestation associated with abnormal placental extracellular matrix deposition and increased decidual vascularization. Our in vitro data suggest that STOX1 suppresses renin through interactions with the 3′-UTR and that renin promoter activity is sensitive to feedback regulation, consistent with knowledge of RAS regulation (47, 48).

Previous reports on STOX1 in pregnancy have focused on gain-of-function studies. One study investigating the transcriptional targets of STOX1, for example, revealed specific patterns of gene expression in STOX1-overexpressing choriocarcinoma cells that mirror those in preeclampsia (27). STOX1 has also been studied using a transgenic mouse model in which placental overexpression of human STOX1 induces a preeclampsia-like phenotype (25). The hypertensive phenotypes observed in both STOX1 gain- and loss-of-function mice are likely explained by the contrasting models. In the transgenic model, supraphysiologic levels of human STOX1 may affect pathways not usually involved in STOX1 signaling or override blood pressure regulatory mechanisms. The severity of the transgenic phenotype is illustrated by the initiation of hypertension in the preimplantation period, blood pressure increases exceeding 60 mmHg, and a 20%–30% reduction in litter size (25). Subsequent studies of the transgenic mouse showed fetal growth restriction (49), cardiovascular dysfunction (24), and postpartum cardiac alterations (50), indicating that this mouse may be a useful model for severe preeclampsia. In contrast, the Stox1-KO phenotype was driven by endogenous upregulation of renin, which would not be expected to have as striking a phenotype because of the presence of intact blood pressure and RAS regulatory mechanisms, and is more consistent with human gestational hypertension.

Alterations in the RAS are well recognized in preeclampsia, which is characterized by increased vascular sensitivity to Ang II and AT1 receptor activation (9, 35, 36, 51–54). Although circulating RAS components (produced by the kidneys) are suppressed in preeclampsia (55, 56), the local uteroplacental RAS has been shown to be upregulated and involved in the regulation of placental angiogenesis and blood flow (35, 36). Indeed, a recent study identified renin as the most upregulated gene in human extravillous cytotrophoblasts from severe preeclampsia cases (57). Our analysis of a published scRNA-Seq data set (38) suggests that *Stox1* is expressed in the equivalent cell type in the mouse placenta, SpA-TGCs, and that STOX1 suppresses placental renin in these cells and in the fetal vasculature. These data align with the mechanistic model we have proposed in which loss of STOX1 suppression of placental RAS underlies the hypertensive phenotype in the KO.

The inverse relationship between STOX1 and renin and potential regulation via the renin 3′-UTR raise the possibility that STOX1 indirectly fine-tunes renin levels by regulating factors that posttranscriptionally repress gene expression. Posttranscriptional regulation of renin has long been recognized and is thought to involve microRNAs (miRNAs) (37, 58). Prior reports suggest that miRNAs target renin in the kidney (59) and the placenta (60), and the human renin 3′-UTR contains a handful of predicted miRNA target sites (61). Whether STOX1 regulates miRNA expression is an outstanding question.

Of hundreds of missense variants in *STOX1*, Y153H had the highest population frequency. These data show that Y153H is tolerated, which would be expected for an inherited susceptibility allele, given that deleterious variants are selected against and thus depleted in the population (46). Indeed, most common conditions are affected by huge numbers of variants and influenced by both genetic and environmental factors (62–64). *STOX1* Y153H could represent a smaller effect size, common variant — one of many that each confers a small increase in disease risk (62, 64). Prior studies examining the frequency of Y153H in preeclamptic versus non-preeclamptic pregnancies have reported conflicting results, with most concluding that the variant is not linked to preeclampsia (20, 22, 23, 65). Neither these studies nor ours, however, addressed potential, important interactions between *STOX1* and other genes. For example, a variant in *NODAL*, a member of the transforming growth factor–β superfamily required for proper trophoblast differentiation (66–68) and fetal growth in mice (69), resides in the same linkage area as *STOX1* and segregates with Y153H in familial preeclampsia (70). A reduction in decidual Nodal results in upregulation of *Stox1* in the mouse placenta and in human trophoblast culture (70, 71). Another example of a STOX1 Y153H-interacting partner is the adhesion protein encoded by CTNNA3, which negatively regulates trophoblast invasion (72). These studies demonstrate the complex biology of STOX1 Y153H at the materno-fetal interface.

The use of a genetic loss-of-function mouse model is invaluable for determining the function of incompletely characterized genes and identifying mechanisms involved in hypertensive disorders of pregnancy (7, 8, 73–75). This analysis provides functional data supporting the hypothesis that mutations affecting STOX1 function may predispose women to developing preeclampsia. We acknowledge that because we studied a whole-body KO, the phenotype cannot be definitely attributed to changes solely in placental STOX1. A limitation is that maternal loss of function was not compared with isolated fetoplacental loss in this study. Thus, it remains uncertain whether *Stox1* loss in maternal versus fetoplacental tissues alone would have produced the same phenotype. Dissecting these complex materno-fetal interactions will require tissue-specific loss-of-function experiments and a broader analysis of the effects of *Stox1* loss on maternal tissues, important next steps for future work. Whether abnormalities in Stox1-KO placentas arose as a direct consequence of STOX1 deficiency, or secondary to renin-induced hypertension or other pathophysiology, is uncertain. With respect to *Stox1*-expressing cell types, comparison of immunohistochemistry (E17.5 placenta; Figure 1F) and scRNA-Seq data (E9.5; Figure 4C) showed potential overlap between *Stox1*^+^ cells (e.g., the labyrinth compartment). However, we were unable to directly correlate these results because of gestational age differences and the selective RNA profiling of *Prdm1*^+^ cells in the study by Nelson et al. (38). Finally, while we used ARB therapy to validate the mechanistic underpinnings of our model, ARB and ACE inhibitor therapy are contraindicated in pregnancy (28), limiting the clinical application of this finding.

The identification of genes involved in hypertensive disorders of pregnancy is important not only for providing insights into disease pathogenesis but also for understanding individual disease risk. This is particularly germane given accumulating data showing that hypertension diagnosed in pregnancy is a harbinger for future cardiovascular disease and increased lifetime risk of serious cardiovascular events, including death (76–78). Our results show that STOX1 regulates gestational hypertension through mechanisms involving the RAS and demonstrate the utility of genetic mouse models for uncovering links between genetic variants and pathways implicated in the pathogenesis of hypertensive disorders of pregnancy.

## Methods

### Generation of Stox1^–/–^ mice.

Targeted disruption of *Stox1* by homologous recombination was achieved by transfecting embryonic stem (ES) cells with a targeting construct containing a 1 kb deletion within exon 3. Fragments used for cloning the targeting construct were generated by high-fidelity PCR using a BAC template (clone RP24-550H10). To generate the Stox1 targeting vector, a 4 kb Xho1-EcoR1 fragment (3′ arm) containing part of exon 3 was ligated into a triple-*loxP* vector downstream of the neomycin cassette; a 3.8 kb SalI-XbaI fragment (5′ arm) containing exon 2 and part of exon 3 was ligated upstream. The linearized targeting construct containing a 1 kb deletion within exon 3 was transfected into ES cells (SV129 line). ES cells were selected with G418 and targeted cells identified by Southern blot analysis. Targeted clones were microinjected into C57BL/6 blastocysts. The resulting chimeric mice were bred to WT SV129 to obtain germline transmission of the targeted allele. *Stox1*^+/–^ mice were mated to generate *Stox1*^–/–^ (Stox1-KO) and WT littermate control mice. Genotyping primer sequences are provided in Supplemental Table 2.

### Mouse breeding and losartan treatment.

At 8 weeks of age, homozygous Stox1-KO or WT crosses were maintained to produce pregnant mice with Stox1-KO or WT litters, respectively. The vaginal plug day was set as E0.5. For mice receiving losartan therapy, 250 μg of losartan (MilliporeSigma) was injected intraperitoneally daily from E14.5 until sacrifice.

### Blood pressure, urinary protein, and placenta biomarker measurements.

Blood pressure was measured using a programmable tail-cuff sphygmomanometer (SC-1000, Hatteras Instruments) as previously described (73). Urine albumin and creatinine levels were estimated using the QuantiChrom BCG Albumin Assay Kit (BioAssay Systems). Serum PlGF and sFlt-1 levels were measured using Quantikine ELISA kits (R&D Systems, Bio-Techne).

### Histology and immunostaining.

Organs collected at E17.5 were fixed, embedded, and sectioned for H&E, immunohistochemistry, and immunofluorescence. For the placenta, H&E-stained cross sections from the midportion were analyzed for morphology and architecture.

Tissue hypoxia was evaluated with a Hypoxyprobe-1 Kit (Chemicon International). Mice were injected intraperitoneally with hypoxic probe (60 mg/kg body weight), 3 hours prior to sacrifice at E17.5. For hypoxyprobe experiments, the probe was detected by immunohistochemistry using the HistoMouse-MAX Kit (Zymed Laboratories, Thermo Fisher Scientific).

For Stox1 and renin immunohistochemistry, deparaffinized sections were treated with warm proteinase K for antigen retrieval before blocking. Immunohistochemistry was performed using a VECTASTAIN ABC Kit and DAB peroxidase substrate reagent (Vector Laboratories). The following primary antibodies and dilutions were used: Stox1 (Santa Cruz Biotechnology, catalog sc-133268, 1:100) and renin (Aviva Systems Biology, catalog ARP41409-T100, 1:100).

For immunofluorescence, frozen sections were fixed with acetone, blocked, incubated with primary antibodies for 1 hour at room temperature, and then labeled with secondary antibodies. The following primary antibodies and dilutions were used: CD31 (BD Pharmingen, catalog 553369, 1:200), laminin (MilliporeSigma, catalog L9393, 1:200), nidogen-1 (Chemicon International, MAB1946, 1:100), and collagen type IV α1 chain (ref. 79, 1:100). Secondary antibodies used were FITC-conjugated goat anti-rabbit IgG (Jackson ImmunoResearch Laboratories, catalog 111-095-144), goat anti-rat (Invitrogen, Thermo Fisher Scientific, catalog A11007), and goat anti-rabbit IgG (Invitrogen, Thermo Fisher Scientific, catalog A11012) conjugated to Alexa Fluor 594. Quantification of CD31-positive vessels was performed by counting the number of vessels per high-power field (3 visual fields per placenta).

### RNA in situ hybridization.

Frozen sections (10 μm) were fixed with 4% paraformaldehyde in PBS. In situ hybridization was performed as previously described (73) using nonradioactive DIG-labeled system (Roche). Stox1 and renin probes were generated by RT-PCR using mouse placental RNA and confirmed by DNA sequencing.

### Transmission electron microscopy.

Kidney tissues were fixed with 2% glutaraldehyde in 0.1 M cacodylate buffer. Kidney segments were processed for electron microscopy and viewed with an FEI Tecnai G2 BioTwin transmission electron microscope or a JEOL 6390 scanning electron microscope as previously described (80).

### Western blotting.

Protein lysates (mouse placenta or HTR-8/SVneo cells) were denatured with SDS sample buffer and heat (95°C for 5 minutes). Samples were separated by 12% SDS-PAGE and transferred to PVDF. Membranes blocked with 5% milk were incubated with primary antibody overnight, then washed and incubated with an HRP-conjugated secondary antibody (Promega, catalog W4011). Bands were detected with the ECL detection system (Pierce, Thermo Fisher Scientific) and quantified using ImageJ (NIH). The following primary antibodies and dilutions were used: renin (Aviva Systems Biology, catalog ARP41409-T100, 1:500) and β-actin (MilliporeSigma, catalog A2066, 1:1000).

### RT-PCR.

RT-PCR was performed using SuperScript II RT, oligo-dT primers (Invitrogen, Thermo Fisher Scientific), and RNA extracted from placenta or cell culture. PCR products were purified and confirmed by sequencing. Bands were quantified using ImageJ. PCR primer sequences are provided in Supplemental Table 2.

### Single-cell RNA analysis.

The published single-cell data set from Nelson et al. 2016 (Gene Expression Omnibus accession GSE74406), which clustered 78 samples of individual cells into 6 cell populations, was analyzed to identify and classify *Stox1*^+^ cells (38). Among *Stox1*^+^ cells, log_2_ expression values were analyzed for *Stox1*, placental cell markers defined in the original study, and renin-angiotensin pathway members. Figure 4A was created with BioRender.com.

### Luciferase assays.

HTR-8/SVneo cells (ATCC) transfected with siRNA and reporter constructs were harvested 48 hours posttransfection. Luciferase activities were measured using the Dual-Luciferase Reporter Assay System (Promega). Predesigned Stox1 (catalog 4392420, siRNA ID s47704) and control siRNAs were purchased from Ambion. PCR using HTR-8/SVneo genomic DNA with subsequent Sanger sequencing were performed to determine the *STOX1* genotype at amino acid position 153 (primer sequences in Supplemental Table 2).

BAC clone RP11-74C13 (PCR template) and pGL3(R2.1) backbone luciferase vector (Promega) were used for reporter constructs. To generate the human renin promoter construct, the PCR fragment of human renin promoter (–5831/+27) was inserted into the multiple cloning site of pGL3(R2.1). To substitute the 3′-UTR in pGL3(R2.1) with the human renin 3′-UTR, the renin 3′-UTR was amplified by PCR and inserted into Xba-I/BamHI site of pGL3(R2.1). The CMV promoter, obtained by PCR of the pCMV-Myc vector (Clontech), was then inserted into the multiple cloning site of this construct.

### Protein sequence alignment and human single nucleotide variant frequencies.

Mouse (NP_001028432) and human (NP_689922) STOX1 amino acid sequences were compared using the UniProt alignment tool (https://www.uniprot.org). Data on human *STOX1* single nucleotide variants were obtained through gnomAD (v2.1.1; https://gnomad.broadinstitute.org; ref. 46).

### Statistics.

Data are presented as mean ± SEM unless otherwise specified. Two-tailed unpaired *t* test and 1-way ANOVA with Bonferroni’s correction for multiple comparisons were used as appropriate, unless otherwise indicated in the figure legend. Statistical significance was defined as *P* < 0.05. GraphPad Prism software (version 8.4.2) and R were used for analyses.

### Study approval.

All experiments were approved by the Beth Israel Deaconess Medical Center Institutional Animal Care and Use Committee.

## Author contributions

JGP generated and analyzed data and wrote the manuscript. KK designed the study, generated *Stox1*^–/–^ mice, generated and analyzed data, and wrote the manuscript. SBL, MK, JLY, YX, and KME conducted experiments and acquired data. RAK analyzed scRNA-Seq data. VHG performed electron microscopy. RK conceptually designed the study, provided intellectual input, and wrote the manuscript.

## Supplementary Material

Supplemental data

## Figures and Tables

**Table 1:**
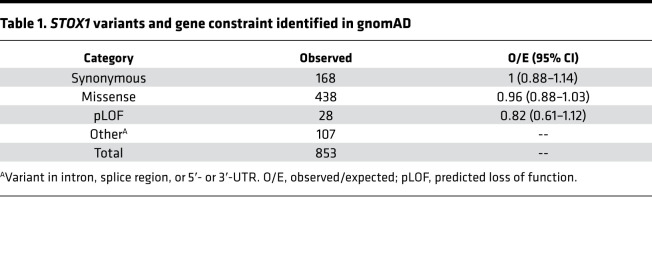
*STOX1* variants and gene constraint identified in gnomAD

## Version 1 12/10/2020

In-Press Preview

## Version 2 01/25/2021

Electronic publication
