# Supplementary material for: STOX1 deficiency is associated with renin-mediated gestational hypertension and placental defects
Source: JCI Insight. 2021 Jan 25;6(2):e141588. doi: 10.1172/jci.insight.141588 (PMC7934881; doi:10.1172/jci.insight.141588)
Supplement: Supplemental data [file jciinsight-6-141588-s047.pdf]

Supplemental Figure 1.

(A) Southern blot analysis of targeted ES cell clone used to generate Stox1 KO mouse.

(B) PCR genotyping of Stox1 mice. WT allele, 559 bp; KO, 448 bp.

(C) Tissue expression of *Stox1* and β-actin by RT-PCR.

Supplemental Figure 2.

(A) Immunofluorescence for nidogen-1 (AKA entactin) and Col IVα1 in the placenta. Scale bar: 50 μm. D, decidua; JZ, junctional zone; L, labyrinth. Solid line marks border between the decidua and junctional zone; dotted line marks border between junctional zone and labyrinth.

(B-C) Expression of genes involved in hypertension, vascular integrity, and inflammation in the placenta (RT-PCR) reveals increased renin mRNA.

(D) Renin expression increased in Stox1 KO placenta but not Stox1 heterozygotes. *Stox1* genotypes of female and male mice shown. F, female; M, male.

(E-F) Renin expression in the kidney no increased in Stox1 KO by RT-PCR (E) or Western blot analysis (F). Prorenin (44 kD) was the predominant form detected in the kidney.

Supplemental Figure 3.

(A) Expression of markers genes (defined by Nelson et al. 2016) in *Stox1*+ cells. NK, natural killer. SpA-TGC, spiral artery trophoblast giant cell.

Supplemental Figure 4.

(A) DNA sequencing results from HTR-8/SVneo human trophoblast cell line showing that this line contains the STOX1 variant allele, Y153H. *NCBI Reference Sequence: NG_012975.2, Homo sapiens storkhead box 1.

Supplemental Table 1: Comparison of systolic blood pressure (mmHg) between groups.

	Gestational time point				
Group	Non-pregnant	E13.5	E14.5	E17.5	Postpartum
WT	116 ± 10	111 ± 9	107 ± 12	109 ± 9[A]	--
Stox1 KO	108 ± 10[A]	114 ± 8	116 ± 12	124 ± 10[B]	109 ± 9[C]
Stox1 KO + Losartan	--	--	116 ± 3	106 ± 9[C]	--

Data are mean ± SD. WT, wild type; KO, knockout; E, embryonic day.

The following numbers of mice were used (group, n):

Non-pregnant: WT, 8; KO, 12

Pregnant E13.5: WT, 10; KO,16

Pregnant E14.5: WT, 9; KO, 14; KO + Losartan, 3

Pregnant E17.5: WT, 5; KO, 15; KO + Losartan, 7

Postpartum (day 10): KO, 7

Statistical significance determined using one-way ANOVA with Bonferroni correction.

[A]Not significant when compared with non-pregnant WT.

[B]Significant difference when compared with non-pregnant Stox1 KO (adjusted $P \leq 0.01$).

[C]Significant difference when compared with E17.5 Stox1 KO; adjusted $P \leq 0.01$ for E17.5 Stox1 KO + Losartan, adjusted $P \leq 0.05$ for postpartum Stox1 KO.

Supplemental Table 2: PCR primer sequences

	Forward	Reverse
**Genotyping**		
Mouse	TTGCAGCAGCTCACATGCAGAG	GCGTTGGCTACCCGTGATATTG (WT allele)<br>GAGGTCCTTAGAGTCGCACATG (KO allele)
Human (HTR-8/SVneo)	TTAGGTGATGTCTTTCCAGTGC	CCAAACACATACAGTGAAGTACAAATAG
**Mouse, RT-PCR**		
ET1	AACTCAGCACCGGAGCTGAGAATGG	CACTTCTTTCCCAACTTGGAACAGGG
COX-2	ACCGTGGGGAATGTATGAGCACAGG	CAGGTCCTCGCTTATGATCTGTCTTG
TNFα	CTTGGGCAGATTGACCTCAG	GTCTCAGCCTCTTCTCATTC
iNOS	CTGATCTTGTGTTGGAGGTG	TCTGCCAGATGTGGGTCTTC
ENG	CAGGTCTCGCAGAAAGAGTC	ACTGGAGGACGATGCTTTGG
Renin	CCGCTACCTTTGAACGAATC	CTGAACCCGTGTCAAAGATG
AGT	AGCTAGAGGATGAGGACCGGAAGCG	AGTGGCAAGTTCATCTTCCACCCTG
ACE	GAGTCTGACAACCTGGAGCAAGACC	CACAGAGGTACACTGCTTGATCCTG
ACE2	GCAATGATGAATCAGGGCTGGGATG	TTCAGATGCTTGGGGGTAGCTGCAG
Stox1	CCCACATCTGTGTGAACCCC	CATTAGGGCAGTGTGCTTGG
β-actin	TGGGCATTGTTACCAACTGGG	AGTTTCATGGATGCCACAGG
**Human, RT-PCR**		
Renin	GAACGAGGTGTGGACATGGCCAGGC	GCTGGAGGAATCCGAAGCATCGAAG
Stox1, primer 1	TGTGTACAGGCCTCAGCACCTGCTG	CTGTCCCTTCCAAGTGACTGTGTTC
Stox1, primer 2	GATTACTATAGCGCAAGAAAAGCC	TTATTAGATCCCAGACTCTGTGTC
β-actin	ACCACACCTTCTACAATGAGCTGCG	CGACGTAGCACAGCTTCTCCTTAATG
**Luciferase constructs**		
Renin promoter	TATGAGCTCTAGCTGGTCTGTGTACAGAGCTAAGC	ATACTCGAGGCTCTCTCTGAGATCCACTGAGGTTC
Renin 3'UTR	ATATCTAGAATTGGCTTCGCCTTGGCCCGCTGAGG	ATAGGATCCCGCAGCCCCTCCCTCTGTTCTAAACCC
CMV promoter	ATAACGCGTGAGTTCGAGCTTGCATGCC	ATACTCGAGGCGGGTACAATTCCGCAGC

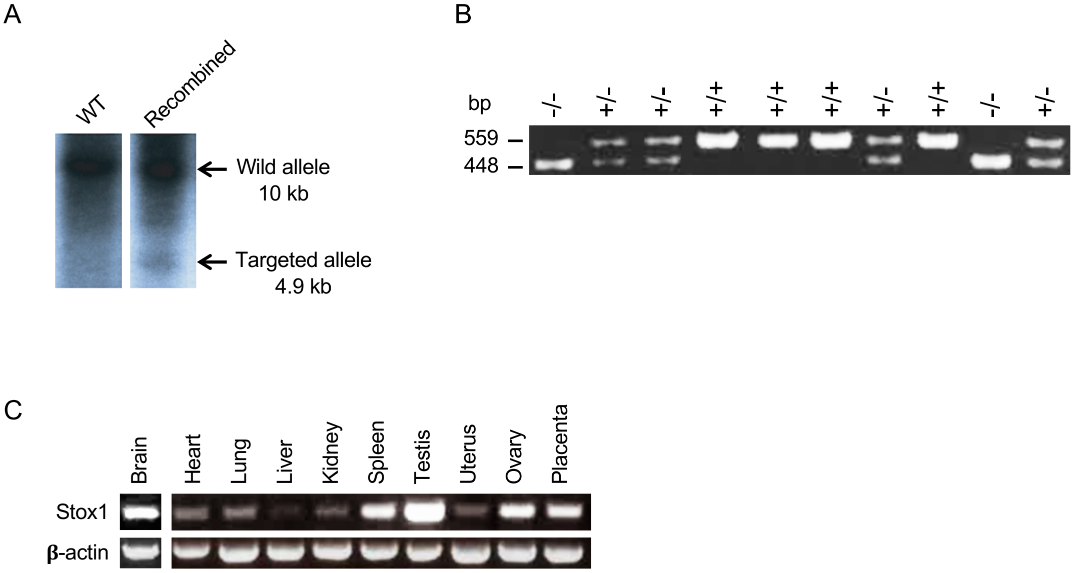

Supplemental Figure 1. (A) Southern blot analysis of targeted ES cell clone used to generate Stox1 KO mouse. (B) PCR genotyping of Stox1 mice. WT allele, 559 bp; KO, 448 bp. (C) Tissue expression of *Stox1* and beta-actin by RT-PCR.

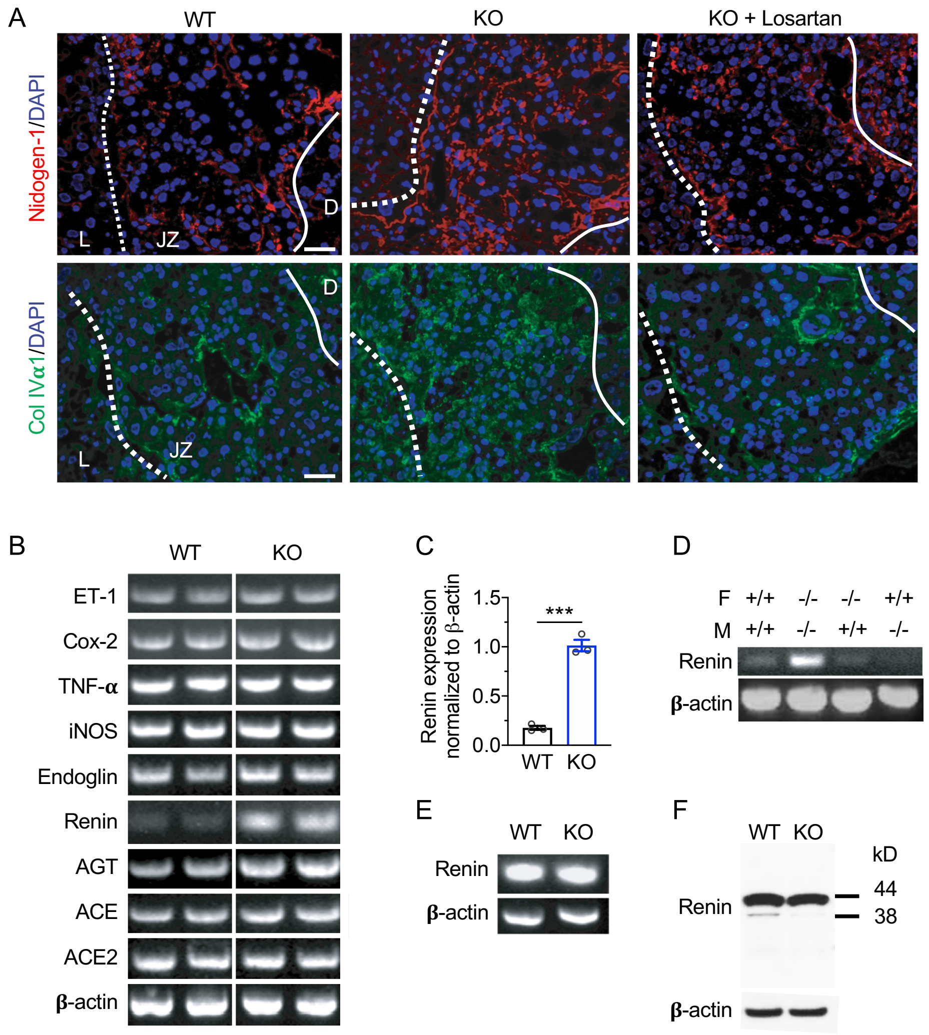

Supplemental Figure 2. (A) Immunofluorescence for nidogen-1 (AKA entactin) and Col IVα1 in the placenta. Scale bar: 50 μm. D, decidua; JZ, junctional zone; L, labyrinth. Solid line marks border between the decidua and junctional zone; dotted line marks border between junctional zone and labyrinth. (B-C) Expression of genes involved in hypertension, vascular integrity, and inflammation in the placenta (RT-PCR) reveals increased renin mRNA. (D) Renin expression increased in Stox1 KO placenta but not heterozygotes. *Stox1* genotypes of female and male mice shown. F, female; M, male. (E-F) Renin expression in the kidney no increased in Stox1 KO by RT-PCR (E) or Western blot analysis (F). Prorenin (44 kD) was the predominant form detected in the kidney.

A

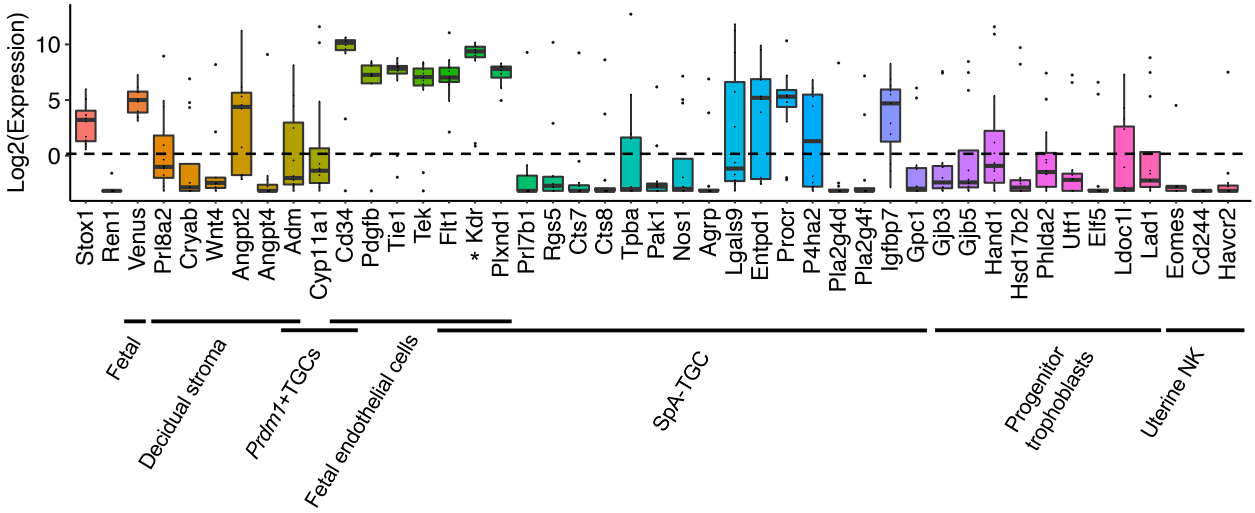

Supplemental Figure 3. (A) Expression of markers genes (defined by Nelson et al. 2016) in *Stox1*+ cells. NK, natural killer. SpA-TGC, spiral artery trophoblast giant cell.

A

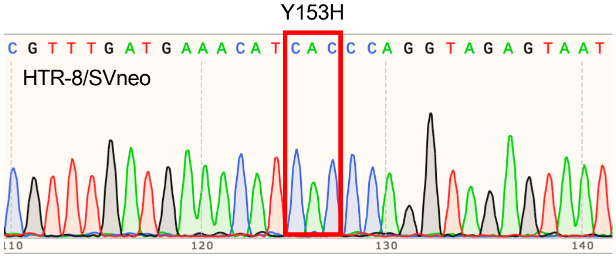

	Genomic DNA sequence	153
HTR-8/SVneo	CGTTTGATGAAACATCACCCAGGTAGAGTAAT	H
Reference*	CGTTTGATGAAACATTACCCAGGTAGAGTAAT	Y

Supplemental Figure 4. (A) DNA sequencing results from HTR-8/SVneo human trophoblast cell line showing that this line contains the *STOX1* variant allele, Y153H. *NCBI Reference Sequence: NG_012975.2, Homo sapiens storkhead box 1.
